# Supplementary material for: Are carbon emissions trading and green financial instruments synergistic? -Comprehensive quantitative research based on content analysis
Source: PLoS One. 2024 Mar 7;19(3):e0298601. doi: 10.1371/journal.pone.0298601 (PMC10919601; doi:10.1371/journal.pone.0298601)
Supplement: S1 Data — (ZIP) [file pone.0298601.s001.zip › Data/地级市Z_F工作报告.zip!/╔╜╢1⁄2/▒⌡╓▌╩╨/└┤╫╘╣1⁄2╓┌║┼í╛┬φ┐╦╩2╛▌═°í┐_2018.docx]

各位代表：

现在，我代表市人民政府向大会报告工作，请予审议，并请市政协各位委员提出意见。

一、2017年工作回顾

过去一年，面对一系列新情况、新问题、新要求、新考验，全市上下在市委的坚强领导下，全面贯彻党的十八大、十九大精神，旗帜鲜明讲政治，扑下身子抓落实，经济社会发展取得了新成效。预计地区生产总值实现2680亿元、增长5.5%；一般公共预算收入226.1亿元、增长5.4%，其中税收收入171.5亿元、增长11.8%，税收占比75.9%、提高3.7个百分点；进出口总额突破百亿美元大关、增长12.6%；粮食总产量355.6万吨、增长16.1%；城镇、农村居民人均可支配收入分别增长7.5%、8%。

（一）着眼全局、攻坚克难，完成宏观调控任务实现稳中有进。2017年，我们面临钢铁去产能、清理整顿电解铝、煤炭消费压减、错峰生产等硬任务，经济发展面临着巨大压力。近年来，我们主动整改钢铁、电解铝发展中存在的问题，为清理整顿工作赢得了主动。一年来，全市各级讲大局、谋长远，行动坚决，完成了省定钢铁、电解铝压减关停任务，基本完成了煤炭消费压减任务，对6类、226家企业实施了错峰生产。同时，加快推进省委、省政府“1+5”重点工作，持之以恒优化营商环境，千方百计为企业提供服务，实体经济实现了又好又快发展。五大千亿级产业集群不断壮大，主营业务收入突破8000亿元，增长13%以上，占到全市规模以上工业的80%以上，真正成为了支撑滨州经济发展的“四梁八柱”。财政消化历年积存问题13.4亿元，同口径增长可达11.6%。北海经济开发区一般公共预算收入突破15亿元，惠民县、阳信县超过10亿元，实现了地方财政收入的历史性跨越。各项调控任务的完成，不仅化解了长期积累的历史问题，而且改善了加快新旧动能转换的宏观环境，为经济稳定健康发展奠定了坚实基础。

（二）抢抓机遇、积极争取，集中办成了一批事关滨州长远发展的大事。滨州港国家一类开放口岸，2017年12月正式获得国务院批准，实现了滨州对外开放的历史性跨越；滨州海事处撤处设局，海域管辖面积由300平方公里扩大到1500平方公里，实现了对滨州港的海事管辖全覆盖，解决了港口和航运发展的瓶颈制约。我市被列入全省高端石化产业“一基地四区”规划，为化工产业高端集约发展创造了新条件。阳信德大铁路至魏桥工业园专用线主线贯通；胶济线接轨至邹平货运铁路正式开工。滨州黄河四桥工可报告获得国家黄委批复。高端铝产业集群被科技部纳入2017年度国家创新型产业集群试点。这些大事的集中推进，奠定了长远发展的基础，积蓄了追赶超越的后劲。

（三）精准发力、创新实干，重点工作实现了新突破。粮食产业创造了“滨州模式”，全国加快推进粮食产业经济发展现场会在滨州召开，我市被授予“全国粮食产业经济发展示范市”。提前一年半完成旱厕改造62.5万户，在全省率先实现全覆盖；天然气“镇镇通”铺设燃气主管道317公里，80个乡镇（街道）接通了天然气；煤改气（电）完成7.5万户，完成省定任务的136%。全省节水灌溉现场会在我市召开，节水灌溉面积达到57.8万亩。强化政策措施配套，秸秆禁烧实现“零点火”。全长19.5公里的东北外环一期工程竣工通车，极大缓解了205国道城北段交通压力。滨州大剧院竣工投用。市人民医院西院区、妇幼保健院和中医院新院奠基开工。面对困难、压力，坚持实干兴市，凝聚了干事创业共识，提升了滨州在全省乃至全国的影响力、知名度。

（四）深化改革、扩大开放，进一步释放了发展活力。持续推进放管服改革，公布了第一批29项“零跑腿”和210项“只跑一次”事项，取消、停征涉企行政事业性收费、政府性基金45项，市场主体达到25.8万户、增长11.8%。设立产业基金15支、62.6亿元，整体规模和运营管理水平进入全省前列。动产抵押和股权、专利权质押融资工作全省领先。不动产登记与房产交易实现一体化办理。公立医院综合改革实现全覆盖。学校去行政化和校长职级制改革全部完成。农村集体产权制度改革试点全面推开。在全国首创法治政府建设目标管理系统，市县两级行政执法体制改革顺利完成，“一村一法律顾问”实现全覆盖。滨州工业大数据服务中心上线运行，京东黄河三角洲云计算大数据产业基地一期部分主体完工，华为大数据中心产业园加快建设。电子商务蓬勃发展，建设电商公共服务中心27个、村镇服务站点2091个，交易额1700亿元、增长30.5%。新引进世界500强企业7家，总数达到30家。积极参与“一带一路”建设，370家企业与沿线40多个国家开展了经贸合作。随着改革开放的深入推进，激发了滨州发展的活力和动力，拓展了参与国际、国内两个市场的广度和深度。

（五）强化整改、协调推进，五场攻坚战取得了显著成效。坚决改善生态环境，中央、省环保督察和环保部大气污染防治强化督查、巡查交办问题全部办结，二氧化硫、PM10 、PM2.5平均浓度明显下降；全面实施“河长制”，扎实推进“清河行动”，6个国控断面全部达标。迎接国务院海洋督察，通过国家土地督察。扎实开展脱贫攻坚，投入专项资金2.2亿元、实施项目156个，1.8万建档立卡贫困人口实现脱贫。积极防控金融风险，成功举办第四届资本对接大会，签订项目2883个，签约金额1356亿元；博兴先达股份主板上市，全市5家企业“新三板”挂牌，16家企业在齐鲁股权交易中心挂牌；深入开展“增信贷、降不良、打逃债”行动，立案打击逃废银行债务59起。退役士兵安置和权益保障按期完成“清零”；圆满完成十九大安保任务，鲁冀界荣乌高速滨州公安检查站等集体和个人受到省委、省政府表彰。顺利通过省安全生产巡查，安全生产考核居全省前列，社会大局和谐稳定。面对五场攻坚战，我们直面困难，迎难而上，经受住了考验，进一步提振了信心。

（六）加大投入、改善民生，实打实地为群众办实事、办好事。一般公共预算民生支出263.7亿元，占财政支出的80.1%，成功入选“2017中国地级市民生发展100强”。城镇新增就业3.7万人，农业转移人口市民化5.9万人、城中村城边村原有居民市民化5万人。完成解决“大班额”项目363个、“全面改薄”71个、“足球进校园”67个，新建、改扩建幼儿园50处；我市被确定为全国青少年校园足球改革试验区。居民医疗保险、基本公共卫生服务政府人均补助标准分别由420元、45元提高到450元、50元；城乡低保标准分别由每人每月500元、315元提高到525元、350元。棚户区改造新开工1.1万户、建成9652户，完成省定任务的101%、180%；治理城市违建563.6万平方米，完成老城区28个道路路口拓宽。美丽乡村覆盖率达到63%，列全省第3位。吕艺镇成功获批第二批“全国特色小镇”。西王社区入选首批“全国农村幸福社区建设示范单位”。城市规划展示馆完成升级改造。《滨州通史》出版发行。在十三届全运会上取得历史最好成绩，魏桥、京博、龙福3家企业冠名乒超球队。以人民为中心的发展思想在各级干部中牢固树立，在社会发展中进一步体现。

（七）依法履职、担当作为，政府自身建设进一步加强。自觉肩负党要管党、全面从严治党政治责任，扎实推进“两学一做”学习教育常态化制度化，牢固树立了“四个意识”。严格落实中央八项规定精神，持之以恒纠治“四风”，严厉查处各类违法违纪案件，作风建设更严更实。坚持用制度管人、管事、管权，制定出台政府工作规则，建立完善了财经、审计、招投标、规划管理等8项制度。主动接受监督，办理人大代表建议94件、政协委员提案332件，答复率均达100%；受理市长公开电话10.6万个、市长信箱来信1512封，办结率均达100%；中国滨州门户网站公众参与指数列全省第1位。持续压减“三公”经费，市级财政拨款安排的“三公”经费同比下降15%。市公共资源交易中心完成交易项目1498个，节支增收15.9亿元，被评为“2017年度全国公共资源交易平台整合先进单位”。政府自身建设取得了新成绩，迈上了新台阶。

各位代表！过去一年，各项工作经受了严峻考验，上级要求高、检查严，高速增长阶段积累的问题多，集中整改时限要求紧。我们紧紧抓住影响全局的大事要事，负重奋进，苦干实干，经济社会始终保持了稳中有进、稳中向好的发展势头。一年来，我们聚焦聚力新旧动能转换，经济发展提质增效；聚焦聚力特色主导产业，发展基础不断夯实；聚焦聚力深化改革，内部动力逐步激发；聚焦聚力重大基础设施建设，发展短板日益补齐；聚焦聚力改善民生，共建共享的良好态势逐步形成。成绩来之不易，经验弥足珍贵。这是全市上下坚决贯彻党中央、国务院和省委、省政府决策部署的结果，是市委统揽全局、加强领导和市人大、市政协监督支持的结果，是全市人民群策群力、砥砺奋进的结果。在此，我代表市人民政府，向全市人民、各界人士、驻滨部队和公安干警，向所有关心支持滨州发展的海内外朋友，致以崇高的敬意和衷心的感谢！

总结成绩的同时，我们也充分认识到工作中还存在不少问题。传统产业占比大、创新能力不足、高精尖人才匮乏，新旧动能转换面临严峻考验；过去高速增长中积累了一些问题，生态环境、节能降耗等整改任务重；投资增速下降，特别是民间投资增长放缓；基础设施建设相对落后，与省内及京津冀地区互联互通水平没有明显提升；群众对美好生活的向往日益增长，民生事业还有很多短板。另外，各项检查多，忙于面上事务多，抓落实、抓推进不够；干部作风不实，责任担当不够，服务意识不强，廉政勤政等仍存在一些问题。对此，要保持清醒认识，采取有力措施，认真加以解决。

二、2018年工作安排

2018年是贯彻党的十九大精神的开局之年，是改革开放40周年，是决胜全面建成小康社会、实施“十三五”规划承上启下的关键一年。新的一年，压力与机遇并存。各项宏观调控措施的实施、高质量发展的新要求，滨州发展将面临新考验。同时，一类口岸对外开放，纳入全省“一基地四区”规划，高铁、高速公路、货运铁路建设等将为经济发展注入强劲动力。长期的发展实践启示我们，只要不断增强辩证思维能力，科学把握分析形势，主动应对各种挑战，就一定能办好自己的事情，就一定能实现更大的作为。

今年工作的总体思路是：全面贯彻落实党的十九大精神，以习近平新时代中国特色社会主义思想为指导，坚持稳中求进工作总基调，坚持新发展理念，坚持高质量发展要求，坚持以供给侧结构性改革为主线，按照“六个更加注重”发展思路，聚力抓好新旧动能转换、乡村振兴、城乡建设、基础设施、改革开放、生态文明、改善民生七项重点工作，加快建设更高质量更高水平的小康滨州。

今年全市经济社会发展的主要预期目标是：地区生产总值增长7%左右，一般公共预算收入增长6.5%左右，规模以上工业增加值增长6.5%左右，固定资产投资增长8%左右，社会消费品零售总额增长9.5%左右，进出口总额平稳增长，城镇登记失业率控制在3.6%以内，城镇、农村居民人均可支配收入分别增长7.5%左右和8%左右，人口自然增长率控制在14‰以内。全面完成国家和省下达的节能减排约束性指标。以上预期目标，突出了稳中求进工作总基调，突出了高质量发展的根本要求。稳中求进既是对经济工作的要求，也是治国理政的重要原则。要稳定经济和社会发展基础，在稳的基础上，努力实现有质量、有效益、有一定速度的增长。具体工作中，重点抓好“七个聚力”：

（一）聚力新旧动能转换，壮大五大千亿级产业集群。要围绕高质量发展阶段的新要求，坚持以“四新”促“四化”实现“四提”，实施好新旧动能转换重大工程，重点推动五大千亿级产业集群向高端迈进。五大千亿级产业集群是滨州经济的压舱石、顶梁柱，是转型升级、高质高效发展的主力军、主战场，市、县区各级各部门都要向五大产业倾斜，全力搞好服务支持。要突出规划引领，健全完善五大千亿级产业集群发展规划，强化规划引领政策支持，加快国家级轻质高强新材料产业基地、高端石化产业基地、粮食加工转化基地、新型纺织产业基地、优质畜牧水产基地和军民融合产业园建设，形成高质量、可持续的产业发展格局。要坚持创新驱动，启动国家创新型城市建设，加大技术研发力度，着力突破制约产业发展的核心技术，推进技术升级、产品升级、价值链升级。推进产学研用深度融合，推广魏桥与苏州大学、西王与中科院、滨化与清华工研院等合作模式，扶持各类研发平台建设，拉长产业链条，提高产品档次，实现借梯登高、借力发展。要实施人才强市战略，牢固树立靠科技兴产业、靠人才强企业理念，进一步解放思想，采取各种形式引进、用好人才，努力争取每个产业都有科研合作、每个骨干企业都有专业人才团队。强化知识产权创造、保护、运用，加快滨州知识产权学院建设，打造山东知识产权数字信息中心。要深入推进质量提升行动，大力实施质量强市和标准化战略，引导企业更多参与国际、国家标准化活动，培育更多领军企业、单项冠军产品。要鼓励发展民营经济，全面落实省非公经济“十条意见”，努力营造创新创业的良好氛围。支持区域特色民营经济发展，培植壮大滨州汽车轻量化、博兴金属板材及商用厨具、惠民化纤绳网、阳信餐具等产业，进一步增强区域经济发展活力。要加快服务业发展，发挥万达广场、山东圣豪、京博物流等龙头企业辐射带动作用，促进生产性、生活性服务业加快发展。鼓励各类服务业创新发展、绿色发展，在大数据、共享经济、教育、卫生、养老、文化旅游等领域实现较快发展。要加快各类开发区建设，强化开发区产业发展、项目建设、税收增长主阵地作用，支持开展体制机制创新，配套完善水电路讯等基础设施，搭建产业、项目、人才、资金集聚平台，确保发展质量、速度优于县区、乡镇。规范发展各类园区，落实“进区入园”要求，调整优化产业布局。加快高新技术企业培育，支持信息技术、高端装备、新能源新材料、智慧海洋、医养健康等新兴产业发展，支持高新区创建国家高新区。要持续优化营商环境，想企业之所想，急企业之所急，帮助解决企业发展中遇到的问题。各级干部要对企业真帮实扶，努力构建“亲”“清”新型政商关系。弘扬优秀企业家精神，培养壮大企业家队伍，鼓励企业在政策法规范围内放开手脚、大胆创业。

（二）聚力实施乡村振兴战略，推动农业农村优先发展。要壮大提升粮食加工、畜牧水产两个千亿级产业集群，积极发展农产品精深加工，推动产业链条前端向良种培育、种植养殖延伸，打造规范、稳定的供应链；中端不断提升加工能力、水平；后端向产品销售、品牌推广领域深化，形成高附加值的价值链，实现一二三产融合发展。开展粮食高产创建，提高粮食产量和质量，增加“渤海粮仓”推广面积。发展肉牛、肉羊、肉禽标准化养殖，大力推广粮改饲。加快海洋经济发展，实施标准化生态池塘改造、种业提升和工厂化养殖，加快发展“虾贝虫”经济。要改善提升农业生产条件，扩大“十统一”面积，推广水肥一体化技术，改造中低产田和盐碱荒地。加强水资源管控和水土保持综合治理，推动林水会战提档升级，抓好旱田水源配套，大力发展节水灌溉，加强黄河水资源调度，支持县区争创全国节水灌溉示范县，创建全国水生态文明城市。深入开展国家森林城市创建，新增造林面积25万亩。建立农机农艺融合示范基地，推进秸秆综合利用。实现涉农乡镇为农服务中心全覆盖，提升供销社服务“三农”水平。要深化农村改革，落实农村土地“三权”分置制度，鼓励农民进行土地经营权流转，积极推进适度规模经营。支持有实力的企业进入农业领域，集中发展一批农业合作社、家庭农场。发挥黄河三角洲（滨州）国家农业科技园区、沿黄生态高效现代农业示范区带动作用，扩大优质粮品、蔬菜、瓜果、食用菌等种植面积，提升京津冀“米袋子”“菜篮子”“果盘子”市场份额。要加强农产品质量监管，推行食用农产品合格证和市场销售凭证“双证制”管理，创建省级出口食品农产品示范区、省级农产品质量安全市，加快推进农业由增产导向转向提质导向。

（三）聚力推进城乡建设，打造优美宜居家园。要推进“四环五海”提升工程，围绕原规划，进一步完善提升规划水平，在完成新一轮城市总体规划修编基础上，加快编制控制性详规，推进“城市双修”试点。研究做好“水”的文章，实施城区水系提升工程，解决好外环河水系不通、城区水质不好等问题。规划建设市城区南部污水处理厂，推进中水回用。开展老城区雨污分流改造工程，深度整治张肖堂干渠、秦台河、码堡沟等城区河道，抓好黑臭水体治理。研究做好“绿”的文章，开工建设市植物园，实施街头游园和绿道建设工程，加强城区绿地精细化养护，提升绿化档次和水平，确保通过国家园林城市复审。巩固国家卫生城市创建成果，开展城市违法建设治理，推进城乡环境综合整治，争创全国文明城市。加快棚户区改造，做好老旧小区棚户区改造安置区建设，新开工3.7万套、基本建成1.5万套。加快市人防科普教育体验馆建设，力争上半年开馆试运行。加大调控力度，规范房地产市场秩序，满足市场合理住房需求。要推进美丽乡村“十百千”工程，实现至少10个示范乡镇美丽乡村全覆盖，创建100个市级以上示范村，完成1000个村的提升工作。抓好“四好”农村路建设，开展农村道路“三通”回头看，提升完善配套建设水平，建立管护长效机制。巩固旱厕改造、煤改气（电）工作成效，推进农村危房改造，不断提升群众生活质量。

（四）聚力重大基础设施建设，进一步增强发展后劲。要持续推进港口开发建设，建成两个3万吨级液化码头，推进5万吨级航道前期相关工作。完成通关服务中心、边防检查站营房等口岸查验设施规划建设，早日实现对外开放运营，年内实现吞吐量3000万吨。稳步推进小清河复航，构建海河、港铁多式联运格局。要突出加快铁路建设，做好济滨高铁前期工作，力争年内开工。配合做好京沪高铁第二通道相关工作。加快阳信德大铁路至魏桥工业园专用线配套建设，近期实现运能。集中加快邹平铁路专用线、滨港铁路二期、黄大铁路建设，济青高铁按期完工，北海经济开发区货运通道建成投用。加快滨州港中海沥青输油专线和其他管线建设，建成滨州港首站。要加快公路网建设，秦滨高速埕口至沾化段、长深高速高青至广饶段、济青高速改扩建工程完成年度任务。沾临高速、沿海高等级公路滨州段开工建设。滨海路、渤海二十一路北延工程实现贯通。要抓好滨州黄河四桥建设，加快前期工作，力争年内开工，早日打通向南通道，实现主城区跨河发展。

（五）聚力深化改革开放，努力营造良好内外环境。要全力完成重点改革任务，按照上级部署，在统筹推进放管服、事业单位、盐业、价格等各项改革基础上，扎实做好重点领域改革攻坚。推进国有企业改革，坚持有进有退，优化调整国有资本出资企业结构，实现市属经营性国有资产集中统一监管。推进农村集体产权制度改革，加强土地流转管理服务，完善农村产权交易市场体系。推进统计管理体制改革，做好第四次经济普查。推进医药卫生体制改革，建立现代医院管理制度，加快实施分级诊疗制度，实现公立医院和基层医疗卫生机构医联体、医共体全覆盖。要提升对外开放水平，对接融入“一带一路”和京津冀一体化战略，鼓励企业开展国际产能合作、海外并购，开设海外生产线和产业园，扩大产品出口和先进装备、技术、资源进口，形成优进优出的开放型格局。加快发展外贸综合服务平台、跨境电商、海外仓等新兴业态，培育出口支柱企业。要强化招商引资，发挥省级以上开发区招商引资主战场作用，围绕优势产业，依托专业协会和各类园区，加大对京津冀、长三角、珠三角和国企央企招商引资力度。要防范各类风险隐患，强化金融单位、监管部门、地方政府各自责任，引导金融机构加大支持实体经济力度。鼓励骨干企业利用股市、债市开展多层次资本市场直接融资，优化地方银行资产结构。持续推进“增信贷、降不良、打逃债”行动，严厉打击恶意逃废银行债务行为，切实维护区域金融生态环境稳定。科学管控政府债务，严控政府性债务风险。

（六）聚力生态资源保护，坚决守护碧水蓝天净土。要以铁的决心抓好中央环保督察反馈问题整改，举一反三、狠抓落实，建立环境保护长效机制。实行排污许可证制度，确保主要污染物排放总量继续明显减少。加强源头管控，探索环评管理新模式，提高环保科技化、职业化水平。加快环保机构监测监察执法垂直管理制度改革，做好省级审批权限下放承接。严格环保执法，实施环境信用评价管理，建设智慧环保平台，形成市县乡村四级联网的在线监测、在线监督、在线指挥监管体系，始终保持环境执法高压态势。要大力推进污染防治，全面推行排放许可证制度，组织实施空气污染情况分析，对重点领域、重点环节实行减排限排。严格危险废物管理。扎实做好煤炭消费压减。支持阳信、无棣等县区发展生物质燃料。全面落实“河长制”，加大对省市河流断面监控，确保水质稳定达标。要加强国土资源管理，强化国土管理保护、开发、利用，严格土地执法，依法管好用好土地。充分发挥优势，扩大增减挂钩规模，抓好未利用地开发，用好闲置土地，盘活土地资源。强化耕地数量、质量、生态“三位一体”管护，巩固永久基本农田划定成果。

（七）聚力保障改善民生，切实增强群众获得感。要加大脱贫攻坚力度，今明两年抓好巩固提升，2020年全面完成脱贫任务。进一步完善保障政策，增加扶贫投入，强化精准举措，提升脱贫质量，探索建立贫困人口动态管理、精准帮扶、精准脱贫长效机制。扎实推进黄河滩区迁建，力争三年任务两年完成。要办好人民满意教育，坚持立德树人，加强师德师风师范建设，用心做教育、用心教学生。启动第三期学前教育计划，加快普及高中阶段教育，规范民办教育，发展特殊教育。深化产教融合、校企合作，健全现代职业教育体系。持续推进“全面改薄”，研究解决“大班额”后续工作，全力实施教育教学质量提升工程，努力实现教育优质均衡发展。加快全国青少年校园足球改革试验区建设。积极探索各具特色的课后服务模式，解决学生放学后托管难题。要加强社会保障体系建设，坚持就业优先，城镇新增就业3.55万人，高校毕业生总体就业率稳定在87%以上。实施全民参保计划，落实居民医保、职工医保市级统筹。完善职工基本养老保险基金管理制度，开展职工长期护理保险制度试点，实现全市城乡居民民生灾害救助综合保障全覆盖。要构建多层次养老服务体系，加快老龄事业和产业发展，农村幸福院覆盖60%以上农村社区。强化对已建成各类养老服务实体管理，规范运作、优化服务。鼓励推广“居家养老”等模式。要积极推进健康滨州建设，市人民医院西院区主体封顶，市妇幼保健院和中医院新院完成主体。完善基层医疗卫生机构标准化建设。推进残疾人精准康复服务行动。要深入推进体育惠民工程，广泛开展全民健身主题活动，完成县级以上主城区“15分钟健身圈”建设。完善市民体育健身广场软硬件设施，为广大市民提供功能更加齐全的休闲场所。深化足球试点城市建设，积极承办体育赛事。全力备战省第二十四届运动会。要大力实施文化惠民工程，挖掘优秀传统文化资源，创作文艺精品。抓好文物保护利用和文化遗产保护传承。抓好滨州大剧院运营管理。强化文化市场监管。实现农村（社区）综合性文化服务中心全覆盖。发行“滨州人游滨州”年卡，为市民出游提供便利。要加强和创新社会治理，开展双拥共建，增进军民团结。做好民族、宗教工作，促进民族团结、宗教和谐。深化公共法律服务体系建设，搭建市县乡村四级公共法律服务平台。完善社会治安防控体系，推进综治中心标准化建设和网格化服务管理，深化“一村一警”警务模式，坚决打击各类违法犯罪。严格落实“四个不能省”要求，推动“双重预防体系”建设，加强重点领域专项治理，坚决遏制恶性安全事故发生。

三、从严从实加强政府自身建设

新时代要有新形象，新征程呼唤新作为。要紧贴新任务、新要求，紧贴人民群众对美好生活的向往，努力建设人民满意、群众信赖的服务型政府。

（一）自觉用习近平新时代中国特色社会主义思想武装头脑。深入学习贯彻党的十九大精神，自觉用习近平新时代中国特色社会主义思想武装头脑、指导实践、推动工作。牢固树立“四个意识”，切实增强“四个自信”，坚决捍卫习近平总书记的核心地位和领袖权威，坚决维护党中央权威和集中统一领导。深入开展“不忘初心、牢记使命”主题教育，加强党性修养，提高政治觉悟，激励广大党员干部争做人民公仆、时代先锋。

（二）加大简政放权力度，改进优化政务服务。继续推进放管服改革，深入开展“双随机一公开”监管，构建权责明确、透明高效的监管机制。加大商事制度改革，深化“先照后证”改革，扩大“证照分离”试点，全面实施“多证合一”，加快推进实施企业登记电子化和电子营业执照。建成市级政务服务中心，完善“互联网+政务服务”体系，提升公共资源交易平台功能，整合形成“12345”服务热线，打造政务服务“最多跑一次”品牌。

（三）坚持依法行政，建设法治政府。深化行政执法体系建设，严格落实执法公示、全程记录、法制审核制度，全面规范行政执法行为。完善法治保障体系建设，推动出台公共数据和电子政务、社会信用、科技成果转化、知识产权保护、能源节约及循环低碳经济等领域的法规规章。进一步完善监督体系建设，依法接受人大工作监督和法律监督，自觉接受政协民主监督，主动接受社会和舆论监督；密切与各民主党派、无党派人士和工会、共青团、妇联等群团组织的联系沟通，认真做好人大代表建议、政协委员提案办理。

（四）强化作风转变，树牢廉洁勤政形象。全面落实从严治党主体责任，坚决履行党风廉政建设“一岗双责”。深入开展反腐败斗争，依法严厉打击各类违法犯罪行为。支持国家监察体制改革，实现对公职人员监察全覆盖。全面贯彻党内政治生活准则和党内监督条例，严格落实中央八项规定和实施细则精神，坚决纠正“四风”问题“十种表现”。突出对脱贫攻坚、生态环保、新旧动能转换等重点领域监督，推进公共资金、国有资产、国有资源、领导干部经济责任审计全覆盖。加大政府系统预决算和“三公”经费公开力度，厉行节约、勤俭办事，确保把有限的资金用在刀刃上。

各位代表，滨州已经站在新时代的历史新起点上，让我们在习近平新时代中国特色社会主义思想指引下，在中共滨州市委的坚强领导下，敢于担当，创新实干，为全面建设更高质量更高水平的小康滨州而努力奋斗！
